# Prediction of two month modified Rankin Scale with an ordinal prediction model in patients with aneurysmal subarachnoid haemorrhage

**DOI:** 10.1186/1471-2288-10-86

**Published:** 2010-09-29

**Authors:** Roelof Risselada, Hester F Lingsma, Andrew J Molyneux, Richard SC Kerr, Julia Yarnold, Mary Sneade, Ewout W Steyerberg, Miriam CJM Sturkenboom

**Affiliations:** 1 Department of Medical Informatics, Erasmus MC, Rotterdam, the Netherlands; 2 Department of Public Health, Erasmus MC, Rotterdam, the Netherlands; 3 Oxford Neurovascular & Neuroradiology Research Unit, University of Oxford, Oxford, UK; 4 Department of Neurosurgery, West Wing, John Radcliffe Hospital, Oxford, UK; 5 Department of Epidemiology, Erasmus MC, Rotterdam, the Netherlands

## Abstract

**Background:**

Aneurysmal subarachnoid haemorrhage (aSAH) is a devastating event with a frequently disabling outcome. Our aim was to develop a prognostic model to predict an ordinal clinical outcome at two months in patients with aSAH.

**Methods:**

We studied patients enrolled in the International Subarachnoid Aneurysm Trial (ISAT), a randomized multicentre trial to compare coiling and clipping in aSAH patients.

Several models were explored to estimate a patient's outcome according to the *modified Rankin Scale *(mRS) at two months after aSAH. Our final model was validated internally with bootstrapping techniques.

**Results:**

The study population comprised of 2,128 patients of whom 159 patients died within 2 months (8%). Multivariable proportional odds analysis identified World Federation of Neurosurgical Societies (WFNS) grade as the most important predictor, followed by age, sex, lumen size of the aneurysm, Fisher grade, vasospasm on angiography, and treatment modality. The model discriminated moderately between those with poor and good mRS scores (*c *statistic = 0.65), with minor optimism according to bootstrap re-sampling (optimism corrected *c *statistic = 0.64).

**Conclusion:**

We presented a calibrated and internally validated ordinal prognostic model to predict two month mRS in aSAH patients who survived the early stage up till a treatment decision. Although generalizability of the model is limited due to the selected population in which it was developed, this model could eventually be used to support clinical decision making after external validation.

**Trial Registration:**

International Standard Randomised Controlled Trial, Number ISRCTN49866681

## Background

Prediction research typically aims to predict outcome of individual patients after the onset of a certain disease, using prognostic models. These models, preferably based on data directly available at hospital admission, are essential to support clinical decision making, and to facilitate reliable comparison of outcomes between different patient series and variation in results over time. Furthermore, prognostic models have an important role in randomized controlled trials (RCT), for stratification [1] and statistical analyses that explicitly consider prognostic information, such as covariate adjustment [2,3], and may provide realistic and evidence-based expectations to relatives.

The majority of published prognostic models predicts a binary outcome, such as case-fatality using binary logistic regression [4-7]. Also, outcomes at ordinal scales are often considered as a dichotomized variable. However, there are several objections against collapsing an ordinal outcome scale into a binary one. First, the cut off for dichotomisation is arbitrary and may vary over studies in a single medical field [4,5,7]. Secondly, from a statistical perspective dichotomisation is a waste of information and reduces statistical power for the analysis of treatment effects or other covariates of interest [8,9]. Furthermore, from a clinical point of view dichotomisation may lead to less useful models. For example, for a patient with a minor stroke a model predicting survival versus mortality is of limited value since the risk is low, while a prediction of complete recovery versus some remaining symptoms may be very useful. For a patient with a severe stroke, this will be the other way around.

An alternative for dichotomisation is application of a statistical approach that uses the full ordinal outcome scale. This leads to efficient use of the data and clinically relevant predictions. Several of these approaches for modelling ordinal response variables have been proposed, including proportional odds (PO) logistic regression, multinomial (or polytomous) logistic regression, or simple linear regression [10]. Each of these methods has its pros and cons.

Our aim was to develop an ordinal prognostic model to predict clinical outcome at two months in patients with aneurysmal subarachnoid haemorrhage (aSAH), based on clinical features and neuro-imaging which are regularly readily available on admission to a neurological or neurosurgical unit. SAH is a devastating event, causing substantial mortality. In 85% of the patients, the SAH is caused by rupture of an aneurysm (aSAH) [11,12]. Of those who survive the first month, approximately one third remains dependent with respect to daily activities during the remaining lifetime [11]. Also amongst patients who regain independency, quality of life remains reduced [13]. A frequently used outcome measurement is the modified Rankin Scale (mRS) [14]. This is an ordered scale for measuring motor function and runs from 0 (no symptoms at all) to 6 (dead) (table 1).

**Table 1:** Definition of the modified Rankin scale

Grade	Description
0	No symptoms at all
1	No significant disability despite symptoms; able to carry out all usual duties and activities
2	Slight disability; unable to carry out all previous activities, but able to look after own affairs without assistance
3	Moderate disability; requiring some help, but able to walk without assistance
4	Moderately severe disability; unable to walk without assistance and unable to attend to own bodily needs without assistance
5	Severe disability; bedridden, incontinent and requiring constant nursing care and attention
6	Dead

## Methods

### Patients

Data were collected prospectively by the Medical Research Council funded International Subarachnoid Aneurysm Trial (ISAT) (International Standard Randomised Controlled Trial, Number ISRCTN49866681). All centres obtained local ethics or institutional review board consent before enrolling patients (see Appendix 1). Able patients provided written informed consent. However, some ethics committees allowed assent from relatives to enable patients who could not give their own written consent to be enrolled in the trial. Full details of ISAT are available elsewhere [15]. The aim of the trial was to determine whether treatment using endovascular coiling reduced the risk of patients being dependent or dead at one year by 25 percent (as defined by modified Rankin Scale grade 3-6) when compared with neurosurgical treatment (clipping) for that cohort of patients.

### Predictors and outcome

We considered all patient characteristics that could be obtained easily and reliably within the first hours after hospital admission and that were also present in the ISAT database. These included age, gender, previous occurrence of SAH, CT scan Fisher grading, World Federation of Neurosurgical Societies (WFNS) grading, number of intracranial aneurysms, location of the aneurysm, maximum lumen size of the aneurysm, vasospasm on angiography, and intended treatment at randomization. Fisher grading of blood visible on a plain CT scan runs from grade 1 ("no blood visible") up to grade 4 ("intraventricular or intraparenchymal blood"). WFNS scale runs from grade 1 ("Glasgow Coma Scale (GCS) 15 and no motor deficit") to grade 5 ("GCS 3-6 with or without motor deficit"). One additional grade was created in ISAT for those in whom WFNS could not be assessed; 'grade 6'. The number of aneurysms was dichotomized into one or more than one intracranial aneurysms. Four aneurysm locations were distinguished: Anterior Cerebral Artery (ACA), Internal Carotid Artery (ICA), Middle Cerebral Artery (MCA), and Posterior Circulation (PC). The maximum lumen size of the aneurysm was expressed in millimetres. Vasospasm was examined on angiography and dichotomized into 'absent' or 'present'. Treatment was either neurosurgical clipping or endovascular coiling; we used treatment as allocated by the randomization procedure. The outcome measure in our study was the modified Rankin Scale (mRS) at two months (table 1) [14].

### Model

We started the development of the model discarding patients without information on outcome. The few missing values in predictors were imputed by means of single imputation (SI, in R language: aregImpute, n.impute = 1, type = 'pmm').

A simple approach to analyze an ordinal outcome, such as the mRS, is to dichotomize the outcome variable by one of several possible cut off points, e.g. 01 vs. 23456 [5], 012 vs. 3456 [4,15], 0123 vs. 456 [7], and 012345 vs. 6 (case-fatality) [6]. We applied binary logistic regression to develop models for these dichotomized responses. Next, we addressed the two main aspects of our ordinal outcome; the fact that it contains order and separate categories. A simple solution for modelling order, while neglecting the categorised nature of our outcome, is to apply linear regression using ordinary least squares. For the opposite - modelling categories, while neglecting order - we used multinomial regression. A more sophisticated approach is to use a proportional odds (PO) model. Such a model takes both order and separate categories into account. The PO logistic model is a rather straightforward extension of binary logistic regression [16]. A common set of regression coefficients is assumed across all levels of the outcome, and intercepts are estimated for each level. The advantage of the PO model is its parsimony in dealing with an ordered outcome. The price we pay is the assumption of proportionality of the odds. This assumption is equivalent to saying that any cut-point on the outcome scale would lead to the same (binary) logistic regression coefficient [10].

We inspected proportionality by studying the univariate odds ratios for each cut off for each predictor. We plotted the score residuals of binary logistic models for each potential predictor separately. The trend of the score components against the levels of the outcome scale should be flat if the proportional odds assumption holds [17]. When the PO assumption is not fulfilled for all potential predictors, we could also investigate a further alternative model: the *partial *PO model [18].

The association between predictors and outcome is expressed as odds ratios (OR). Predictors have statistically significant effects when the 95% confidence interval does not include the value one.

A multivariable PO model was developed containing predictors that met Akaike's Information Criterion (AIC) in a backward stepwise procedure [19]. AIC compares models based on how well they fit the data, but penalizes for the complexity of the model. AIC requires that the increase in model χ^2 ^when entering a new predictor has to be larger than two times the degrees of freedom: χ^2 ^>2 *df*. When considering a predictor with 1 *df*, such as gender, this implies that χ^2 ^has to exceed 2, equivalent to p < 0.157. When considering a predictor with 2 *df*, χ^2 ^>4, or p < 0.135; and in case of 4 *df*, χ^2 ^>8, or p < 0.092 [10].

### Performance

The performance of the final PO model was assessed with respect to calibration and discrimination. Calibration is the ability of the model to produce unbiased estimates of the probability of the outcome. Calibration was tested with a goodness of fit test, which assesses agreement between predicted and observed risks over the full range of predicted probabilities. Discrimination is the model's ability to separate patients with different outcomes. To quantify the discrimination, we used the *c *statistic. A model with a *c *statistic of 0.5 has no discriminative power at all, for example a coin flip. A *c *statistic of 1.0 reflects perfect discrimination.

### Model validation

The performance of a prediction model is generally worse in new patients then initially expected. This 'optimism' of the original model can be studied with internal validation techniques [10]. Internal validity of the models was assessed with standard bootstrapping procedures. Bootstrapping involves drawing samples of patients with replacement from the development population. Each sample can be considered as if one is repeating the data collection with the same number of patients and under identical circumstances as the original. Regression models were estimated in 300 bootstrap samples. Each of these 300 models was evaluated on the original sample. The average difference in the *c *statistic was determined to indicate the optimism in the initially estimated discriminative ability [10]. A shrinkage factor was estimated from the bootstrap validation procedure and we shrunk the regression coefficients to provide better predictions for future patients [10].

All statistical analyses were performed using R software, version 2.8.1 (R Foundation for Statistical Computing, Vienna, Austria).

## Results

A total of 2,143 patients were recruited to the ISAT trial by 43 neurosurgical centres, mainly in Europe. We excluded 15 patients with missing information on the two month mRS. Fisher grade of 14 patients was not available and in one patient no information on vasospasm was available. We statistically imputed these missing values, leaving 2,128 patients for analysis, of whom 347 were in mRS grade 0 (16%), 583 in mRS grade 1 (27%), 528 in mRS grade 2 (25%), 296 in mRS grade 3 (14%), 80 in mRS grade 4 (4%), 135 in mRS grade 5 (6%) at the two month assessment, and of whom 159 (8%) died before the two month assessment.

**Figure 1:**
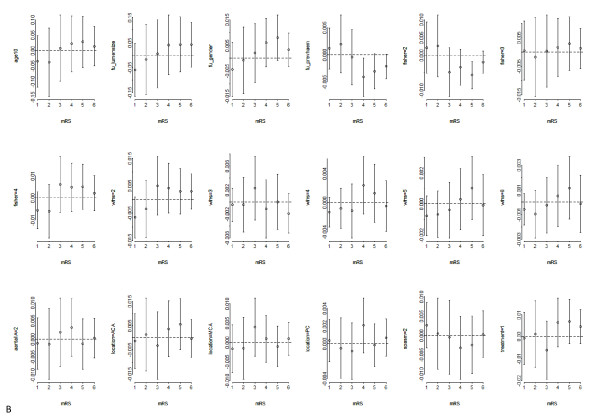
**Residual plots **(**score.binary**) **of univariate associations of potential predictors to examine deviations from the PO assumption**.

Univariate analyses in the binary models for different cut offs, the PO model, and the linear regression model are presented in table 2. The ORs for each cut off were reasonably similar except for previous SAH (fu_prevhaem) and Fisher grade 2 (Fisher = 2). This violation of the PO assumption is also noted by statistically significant deviations from the horizontal line in figure 1. The linear regression coefficients were surprisingly close to the ORs from the PO model. The multinomial model yielded 108 coefficients, apart from 6 intercepts (not shown). In a partial PO model 6 intercepts were fitted, 6 coefficients for previous SAH, 18 coefficients for Fisher grade, and one for each of the other predictors (not shown).

**Table 2:** Univariate associations for different cut offs for mRS (odds ratios), the univariate PO estimate (odds ratios), and linear regression (linear regression coefficient)

	1	2	3	5	PO	linear
**age [10 years]**	1.19	1.38	1.48	1.49	1.26	1.28
**lumensize [mm]**	1.05	1.06	1.08	1.10	1.05	1.06
**Sex**						
Male	0.67	0.71	0.83	0.86	0.69	0.75
**previous SAH**						
Yes	1.19	1.01	0.61	0.55	1.04	0.93
**Fisher grade**						
1	1	1	1	1	1	1
2	0.93	1.39	2.29	2.01	0.99	1.09
3	1.29	2.51	4.45	4.32	1.50	1.67
4	2.03	4.47	7.72	7.31	2.49	2.72
**WFNS grade**						
1	1	1	1	1	1	1
2	1.94	2.47	2.60	2.40	2.08	1.99
3	3.34	4.46	3.67	2.64	3.51	3.18
4	6.74	7.85	12.55	7.84	8.38	7.99
5	6.06	10.83	16.26	12.00	12.43	11.25
(not assessable) 6	5.35	9.92	13.14	9.55	9.97	9.37
**number of IA**						
>1	1.11	1.21	1.30	1.17	1.15	1.15
**location**						
ICA	1	1	1	1	1	1
ACA	0.86	0.83	1.04	0.90	0.86	0.90
MCA	0.80	0.95	1.01	0.97	0.84	0.88
PC	0.89	0.83	1.74	1.39	0.97	1.06
**vasospasm**						
present	1.69	1.65	1.49	1.71	1.67	1.60
**intended treatment**						
Coil	0.69	0.60	0.81	0.88	0.68	0.73

For the sake of interpretability and clinical usefulness, we chose to accept the violation of the PO assumption in the PO model. Age and WFNS grade were the most important predictors in the multivariable PO model (table 3). Other statistically significant predictors were sex, lumen size, Fisher grade, vasospasm, and treatment modality.

**Table 3:** Final PO and linear models - shrunken estimates of OR and regression coefficients.

	PO			Linear		
	OR	**CI**_**min**_^a^	**CI**_**max**_^a^	coefficient	**CI**_**min**_^a^	**CI**_**max**_^a^
**age [10 years]**	1.18	1.10	1.26	1.20	1.13	1.27
**lumensize [mm]**	1.04	1.01	1.07	1.04	1.02	1.07
**Sex**						
Male	0.78	0.66	0.91	0.85	0.73	0.98
**Fisher grade**						
1	1			1		
2	1.00	0.69	1.43	1.07	0.77	1.49
3	1.28	0.91	1.79	1.37	1.01	1.86
4	1.50	1.06	2.13	1.56	1.13	2.14
**WFNS grade**						
1	1			1		
2	1.74	1.44	2.10	1.68	1.42	1.98
3	2.43	1.75	3.39	2.27	1.69	3.04
4	6.04	3.90	9.37	5.70	3.89	8.35
5	7.90	3.43	18.17	7.56	3.72	15.35
(not assessable) 6	7.70	3.91	15.16	7.00	3.92	12.50
**vasospasm**						
present	1.36	1.13	1.64	1.30	1.10	1.54
**intended treatment**						
Coil	0.69	0.60	0.81	0.75	0.65	0.85

OR = odds ratio; CI_min _= lower limit of the 95% confidence interval; CI_max _= upper limit of the 95% confidence interval. ^a ^95% CI was calculated based on the standard error of the estimates of the coefficients in the full model to avoid underestimation of uncertainty.

**Figure 2:**
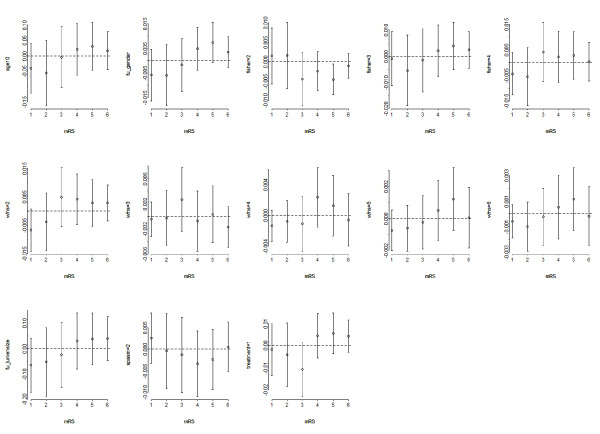
**Residual plots **(**score.binary**) **of predictors in the selected multivariable model to examine deviations from the PO assumption**.

The goodness of fit test yielded a p-value smaller then 0.05 for all levels of mRS, suggesting that the model poorly fitted the data in which it was developed. In our final model the PO assumption was violated only for Fisher grade 2 (figure 2). The *c *statistic of the final model was 0.65 (optimism-corrected: 0.64). Details of the model are described in Appendix 2.

## Discussion

We developed and validated a prognostic proportional odds model to predict the risk of two month modified Rankin Scale in individual patients after aneurysmal subarachnoid haemorrhage. Predictions were based on characteristics that were collected in a large clinical trial and that are regularly readily available on admission to a neurological or neurosurgical unit. The *c *statistic was modest, indicating a mediocre ability to predict clinical outcome at the two month assessment.

The dependence of our proportional odds model on the assumption of proportionality should not be overstressed. The potential inaccuracy caused by mild violation of the PO assumption is likely less severe than would be the case in arbitrary dichotomisation of an ordinal outcome. Dichotomisation involves more loss of information [20]. Probably one would prefer a "wrong, but useful" model, despite possibly violating some underlying model assumptions [21]. Moreover, the PO model predicts the probability of being in each mRS level for each individual patient. This makes the model useful for all patients, regardless of severity.

Besides the PO model we explored several other models. The ordinary least squares model seemed to perform quite well (see table 3). Although the categorical nature of the outcome variable is neglected, the model seems to perform reasonable and may yield estimates of regression coefficients that are quite similar to the PO model. This model might suffice to gain insight in which predictors play an important role in this clinical question. On the contrary, the multinomial model and - to a lesser extent - the partial PO models suffer from highly limited interpretability and therefore usability. A plethora of coefficients is produced by these models. If one is very specifically interested in one outcome grade, the model might be of some use. In most cases, we consider a more pragmatic approach however preferable. There are many more potentially useful modelling techniques for ordinal outcomes [22,23]. One such technique is the continuation ratio (CR) model, which has been said to be likely to fit ordinal responses when subjects have to 'pass through' one category to get to the next. For a worked example see a tutorial by Harrell *et al*. [17].

Several limitations of our analyses should be acknowledged. This study used data from one large trial on a selected population of patients in equipoise regarding treatment with either endovascular coiling or neurosurgical clipping, which limits generalizability. Nonetheless, according to a recently published paper, the ISAT population proved to be quite similar to the population admitted with an aSAH to neurosurgical units in the United Kingdom [24]. The model may perform well in the development sample, but poorly when applied to other groups of patients, for example, a less strictly selected one. Validation of a prognostic model in independent patient series is considered an essential next step [25]. However since large samples of systematically collected data on aSAH are sparse, assessment of external validity is difficult. For now the generalizability and overall validity of our model remains to be established. This will be a topic of future research.

Although our model represents knowledge obtained from 2,128 SAH patients, predictions for individual aSAH patients are always subject to uncertainty. The model makes certain structural assumptions and statistical interaction terms were not included. Hence, it is possible that specific patterns of risk factors are inadequately reflected in the model predictions. Therefore, predictions should be regarded with care and not directly be applied for treatment limiting decisions.

The modest performance of the presented model might potentially be improved by including neuro-imaging biomarkers other than lumen size, location, Fisher grade on plain CT scan, and vasospasm on angiography. Biomarkers regarding anatomy and morphology might be considered, as well as aneurysm characteristics obtained from three and four dimensional angiography [26,27]. Performance may also be improved by inclusion of subsequent information obtained after admission, including temporal course, neuro-imaging at later time points, eventual rebleeding of the aneurysm, delayed ischemic deficit, and other parameters such as hydrocephalus. The objective of the present study, however, was to investigate prognostic models that predict two month mRS with predictors available at admission.

For scientific purposes, we chose to present the final ordinal model as a formula. To increase usability of the model in clinical practice, it could eventually also be presented as a score chart, giving points for the presence of each predictor. The predicted probabilities for each mRS level corresponding to a certain score can subsequently be read from a score plot. Another possibility is to present the model in an Excel sheet or e.g. as a PDA application.

## Conclusion

We presented a calibrated and internally validated ordinal prognostic model for predicting two month outcome after aSAH. Although generalizability of the model is limited due to the selected population in which it was developed, this model could eventually be used to support clinical decision making after external validation in a clinical setting.

## Competing interests

AJM has a consulting agreement with Micrus Endovascular, a manufacturer of detachable platinum coils. AJM, RSCK, and JY have received assistance with travel and meeting expenses from Boston Scientific Corporation. RR, HFL, MS, EWS, and MCJMS report no competing interests.

## Authors' contributions

RR did the statistical analyses and drafted the manuscript. HFL participated in the statistical analyses and drafting of the manuscript. AJM, RSCK, JY, and MS made substantial contributions to acquisition of the data and have been involved in revising the manuscript critically for important intellectual content. EWS conceived of the study, participated in the statistical analyses and helped to draft the manuscript. MCJMS conceived of the study and helped to draft the manuscript. All authors read and approved the final manuscript.

## Appendix 1

International Subarachnoid Aneurysm Trial, ISRCTN49866681

Ethics/IRB committees for the contributing centres for ISAT

UK:

**Oxford (Centre 01) - **Local Oxford Research Ethics Committee: COREC 94.039

**Edinburgh (Centre 02) - **LREC Western General, Lothian Health Board: 1702/93/5/42

**Nottingham (Centre 04) - **Queens Medical Centre LREC: IMH/LE July 95

**Cardiff (Centre 05) - **LREC Heath Park, Cardiff: 96/1470

**Atkinson Morley (Centre 06) - **LREC St George's Healthcare Trust: 96/33/7

**Newcastle (Centre 07) - **Newcastle & North Tyneside Health Authority: 96/173

**Bristol (Centre 08) - **Frenchay Healthcare Trust Ethics Committee: 96/70

**Hurstwood Park (Centre 11) - **East Unit REC, Mid Sussex NHS Trust: 04/97

**Manchester RI (Centre 12) - **Manchester Health Authority REC (Central): CM/97/068

**Manchester, Salford (Centre 13) - **Salford & Trafford REC: 97/074

**King's, London (Centre 14) - **King's College Hospital REC: 1997/0198

**Birmingham (Centre 18)- **Queen Elizabeth Hospital REC: 1998

**Plymouth (Centre 21) - **Plymouth REC, South West Devon Health Authority: 1127

**Glasgow (Centre 22) - **Southern General Hospital REC: EC/99/S/9

**Queens Square (Centre 23) - **NHNN Joint Medical Ethics: 98/N109

**Liverpool (Centre 29) - **South Sefton REC: EC.70.99.M

**Cambridge (Centre 30)- **Cambridge REC: 2000

**Sheffield (Centre 32) - **South Sheffield REC: SS99-164

**Hull (Centre 33)- **LREC Hull Royal Infirmary: 2000

**Belfast (Centre 34) - **Queens University Belfast REC: 140-00-MREC-98-5-73

**Royal London (Centre 41) - **East London & City HA, ELCHA REC: P/00/252/M

**Oldchurch (Centre 42) - **LREC (Barking & Havering): 64 (SC)

Germany:

**Freiburg (Centre 10) - **Albert Ludwigs Universität Freiburg: 207/96

**Mainz (Centre 20) - **Universitatsklinik Mainz: Aug 98

**Dresden (Centre 24) - **Ethik Kommission Universitatsklinik Carl Gustav Carus: EK10199

**Würzburg (Centre 27) - **Ethik Kommission der Medizinischen Fakultät der Universität Würzburg: 53/99

**Augsburg (Centre 28) - **Krankenhaus Zweckverband Augsburg: Jan/99

**Hamburg (Centre 44) - **Ethik Kommission der arztekammer Hamburg: M-247/2000

Switzerland:

**Lausanne (Centre 25) **- Commission d'Ethique de la Recherche de la Faculté de Medicine: F/15/99

France:

Paris (Centre 35) Amiens (Centre 36) Lille (Centre 37) Montpellier (Centre 39)

**Rennes (Centre 40) Sainte Anne (Centre 45) - **Comité Consultatif de protection des personnes dans la recherche biomedicale éthique: 17 May 2000

Sweden:

**Gothenburg (Centre 09) **- Gothenburg University Hospital: 285-94

**Uppsala (Centre 19) - **Uppsala Universitet: 97446

Finland:

**Turku (Centre 16) - **Joint Commission on Ethics of Turku University Central Hospital: 8/1997

Denmark:

**Odense (Centre 26) - **Scientific Ethics Committee of Vejle & Funen: 19990050

Czech Republic

**Prague (Centre 46) - **Charles University Etika Komise: Dec 2001

Canada:

**Montreal (Centre 15) - **CHUM Pavillion Notre Dame Ethics: 09/1997

**Toronto (Centre 17) - **Toronto Hospital Medical Research Directorate: 97/HO62

USA:

**Baltimore (Centre 31) - **Joint Committee on Clinical Investigation: 99-06-18-08

Australia:

**Perth (Centre 03) - **Royal Perth Hospital Ethics Committee: 05/06/004/F57

## Appendix 2

### Details of the Prognostic Model

The probability of an outcome (mRS level) within two months is calculated according to the logistic formula: 1/(1 + exp^-LP^). The linear predictor (LP) takes the form of LP = intercept + regression coefficients × predictor values.

LP for mRS = 0.250 [mRS ≥ 1] - 1.15 [mRS ≥ 2] - 2.25 [mRS ≥ 3] - 3.08 [mRS≥4] - 3.39 [mRS ≥ 5] - 4.11 [mRS ≥ 6] + 0.164 × age - 0.255 × [male] - 0.00445 × [Fisher grade II] + 0.245 × [Fisher grade III] + 0.404 × [Fisher grade IV] + 0.555 × [WFNS grade 2] + 0.889 × [WFNS grade 3] + 1.80 × [WFNS grade 4] + 2.07 × [WFNS grade 5] + 2.04 × [WFNS grade 6, 'not assessable'] + 0.0404 × lumen size of aneurysm + 0.307 × [vasospasm present]- 0.367 [coil].

Coding of the predictors was as follows: age in decades, lumen size in millimetres; all other predictors, 1 if true and 0 if false.

## Pre-publication history

The pre-publication history for this paper can be accessed here:

http://www.biomedcentral.com/1471-2288/10/86/prepub
